# A mirror therapy system using virtual reality and an actuated exoskeleton for the recovery of hand motor impairments: a study of acceptability, usability, and embodiment

**DOI:** 10.1038/s41598-023-49571-7

**Published:** 2023-12-18

**Authors:** Gabriele Abbate, Alessandro Giusti, Luca Randazzo, Antonio Paolillo

**Affiliations:** 1 Dalle Molle Institute for Artificial Intelligence (IDSIA), USI-SUPSI, Lugano, Switzerland; 2 Emovo Care, EPFL Innovation Park, Lausanne, Switzerland

**Keywords:** Rehabilitation, Stroke, Quality of life, Engineering, Biomedical engineering

## Abstract

Hand motor impairments are one of the main causes of disabilities worldwide. Rehabilitation procedures like mirror therapy are given crucial importance. In the traditional setup, the patient moves the healthy hand in front of a mirror; the view of the mirrored motion tricks the brain into thinking that the impaired hand is moving as well, stimulating the recovery of the lost hand functionalities. We propose an innovative mirror therapy system that leverages and couples cutting-edge technologies. Virtual reality recreates an immersive and effective mirroring effect; a soft hand exoskeleton accompanies the virtual visual perception by physically inducing the mirrored motion to the real hand. Three working modes of our system have been tested with 21 healthy users. The system is ranked as acceptable by the system usability scale; it does not provoke adverse events or sickness in the users, according to the simulator sickness questionnaire; the three execution modes are also compared w.r.t. the sense of embodiment, evaluated through another customized questionnaire. The achieved results show the potential of our system as a clinical tool and reveal its social and economic impact.

## Introduction

Stroke is one of the main causes of disability in the world^1,2^, with huge global costs^3^, which especially burden lower-middle income countries^3,4^. Limitation of the hand functionalities is experienced by most of the people affected by stroke^5–7^ and many patients experience long-lasting hand-motor impairments^8^. The social and functional role played by hands in our everyday life^9,10^ makes hand rehabilitation crucial for the restoration of a good patient’s quality of life. Among the many different rehabilitation strategies, Mirror Therapy (MT) proved to have a positive effect on motor function recovery^11–13^. In a MT session, a mirror is placed along the sagittal plane of the patient (i.e. perpendicular to their torso). The patient performs simple motions with the healthy hand, while the impaired hand stays still and hidden behind the mirror. The reflected view of the moving healthy hand tricks the brain into thinking that also the other (impaired) hand is actually moving. Such an illusion stimulates neuroplasticity and induces the recovery of the lost hand functionalities^11^. A similar illusion can be recreated and improved using a Virtual Reality (VR) setup, thus providing patients with an immersive experience capable of stimulating motor learning, motor recovery, and neuroplasticity^14^. VR-based MT approaches are well-tolerated by patients^15^, and are demonstrated to measurably improve the functional abilities of impaired hands^16–18^. Several meta-analyses show that robotics-supported therapy is a viable complement to traditional methods, with comparable results to the dose-matched standard of care rehabilitation^19–23^. Indeed, robotics methodologies offer attractive ways to improve the efficacy of MT^24^. In particular, robots can assist the movements of impaired limbs and reduce the desynchronization of visual and sensorimotor feedback, which negatively impacts the therapy efficacy^25^. The combined effect brought by the application of VR and robotics is promising for clinical research in neuro-rehabilitation^26^, also because it could increase the frequency and duration of the therapy, resulting thus in better outcomes^27^. It is worth noting that easy-to-use and portable systems can be extensively used on a daily basis and, as a consequence, their acceptance and potentially their efficacy can increase^28,29^. Nevertheless, the use of robotic rehabilitation devices remains limited^30^. To the best of our knowledge, in the specific context of MT, wearable robotics and VR have been combined only in a recently approved clinical trial, whose results are not available yet^31^.

**Figure 1:**
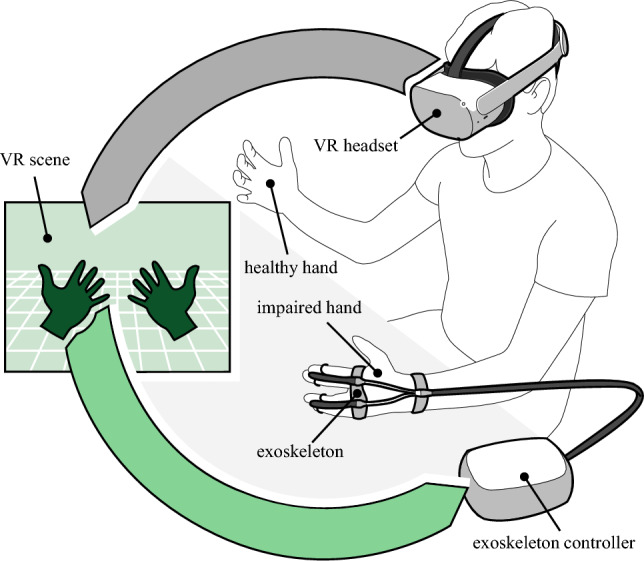
The concept of our work: in VR, the motion of the healthy hand is tracked and mirrored onto the impaired hand; the configuration of the virtual mirrored hand is then reproduced onto the real impaired hand through the exoskeleton.

We propose a system that uses VR and actuated exoskeleton to improve the state-of-the-art of MT. Our system is sketched in Fig. 1. We aim at augmenting the brain illusion of impaired hand motion by coupling visual and physical perception. The former is obtained by mirroring the healthy hand motion onto the impaired one in VR; the latter is obtained by inducing the mirrored motion to the real hand through the action of a soft actuated exoskeleton. We aim to leverage this paired visual-motor effect to produce a sense of embodiment of the virtual hand. We have specifically designed our system using components that are affordable, lightweight, and safe enough for independent domestic use. In particular, the exoskeleton that we adopt is soft, simple, moves slowly, and is compliant with the user’s hand. On the one hand, these specifications meet the requests of stroke patients who have stiff hands and, thus, difficulties in wearing heavy and hard exoskeletons. On the other hand, these requirements set important hardware limitations and implementation challenges that we overcome by testing and comparing several design choices.

The contributions of this work are (*i*) the design and implementation of a MT system that leverages VR and actuated exoskeleton technology; and (*ii*) the tests with healthy users measuring the impact of our design choices on the system’s usability, acceptability, and sense of embodiment. Our tool has social and economic impacts at different levels. As individuals, patients and therapists can directly benefit from our system. At the country level, the national healthcare systems can use our system to elaborate a virtuous rehabilitation procedure. Globally speaking, a cheap and portable tool could reduce disparities with lower-middle-income countries. The system has also the potential for applications in telemedicine, opening interesting and promising scenarios for the future of robotic-assistive rehabilitation.

**Figure 2:**
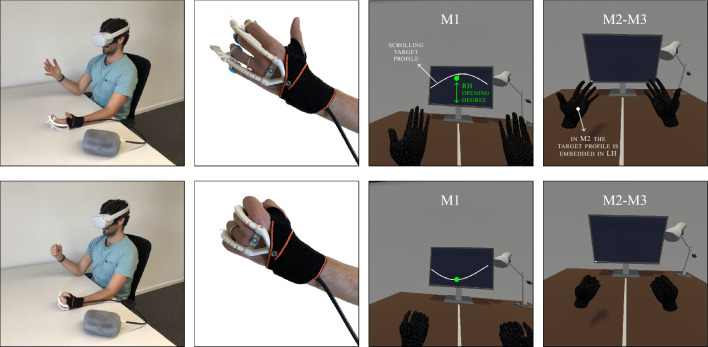
The proposed MT setup: external view (first column), zoom on the exoskeleton (second column), VR scene in M1 (third column) and M2-M3 modes (forth column), when the user opens (first row) and closes the hand (second row).

## Results

The system is evaluated with 21 healthy users (17 M, 4 F, average age of 34.2 years and standard deviation of 8.4 years) having little to no experience with VR and wearable exoskeletons, who volunteered to participate in our user study. The participants are asked to wear the VR headset and the exoskeleton at Left Hand (LH) as if it was the impaired hand in a real rehabilitation setup; the users are told to keep Right Hand (RH) free as if it was the healthy hand (see Fig. 2). A scalar measure representing how much one hand is open is called hereafter *opening degree*. This quantity is measured from the hand-tracking system of the VR headset and its technical derivation is discussed in Methods. The system is tested in 3 operating modes differing in the following aspects: (*i*) which hand is tracked, i.e., RH only or both; (*ii*) how LH is visualized in VR, either by mirroring RH or following a target profile, defined in advance; (*iii*) how the exoskeleton is actuated, i.e., in open-loop fashion following a target profile, or in closed-loop trying to zero the difference between the opening degree of RH and that of LH; (*iv*) how the users are instructed to move RH, which can be done arbitrarily, in accordance with a target profile, or following LH. The single modes are described in detail as follows:

- Mode 1 (M1): the users open and close RH in order to match a moving sine target profile visualized in front of them in VR, see Fig. 2 (third column). The current state of RH is visualized as a green circle, which moves up and down according to the measured RH opening degree. Such a circle serves as online feedback to better regulate the distance from the desired profile: the objective of the user is to open and close RH in order to keep the green circle on the sine profile. In this mode, the users tend to focus their attention on the target profile, while the hands remain in their peripheral vision. The LH opening degree is mirrored from RH and the exoskeleton is directly actuated by the same sine profile used for the visualization in VR.
- Mode 2 (M2): the LH opening degree is not mirrored from RH. In the VR scene, LH is opened and closed according to a target opening degree that has been decided in advance. Users are instructed to follow the movement of the virtual LH with their RH. In this mode, the user focuses their attention on the hands (see the fourth column in Fig. 2). The exoskeleton is actuated to directly follow the same opening degree used for the LH visualization in VR.
- Mode 3 (M3): the users move their RH at their own desire and pace. The LH opening degree is mirrored from RH. The exoskeleton is actuated in a closed-loop fashion to minimize the difference between the opening degree of the real RH and that of the real LH. In this mode, as in M2, users tend to focus on their hands (as shown in the fourth column of Fig. 2).

The working conditions of the 3 modes are summarized in Table 1 and shown in the video contained in the Supplementary Information. The design of these modes is the result of a careful evaluation of the MT context and obtained by practical considerations collected during preliminary tests. M3 is the mode that best resembles the traditional MT setup, where the healthy hand moves according to the user’s will, and the impaired hand motion is perceived as the effect of the mirroring. However, at the beginning of the experimentation, users had difficulties accommodating their own RH motion to the characteristics of the exoskeleton, which is designed to be slow and compliant as it was worn by patients. For this reason, we have designed M1 and M2 where the motion is not arbitrarily dictated by the user, but imposed by slow periodic target profiles (having a period of about 7 s) that comply with the specification of the exoskeleton. More in detail, in M1 the user has to follow a sine profile visualized in front of them, while in M2 the target profile is embedded as an animation of the virtual LH. We have specifically designed these two different strategies to validate one further aspect: how the distraction element impacts the embodiment and the acceptability of the system. In fact, in M1 the users focus on the profile visualization, and the hands stay in their peripheral view, while in M2 they point their gaze at the hands (cf. third and fourth column in Fig. 2).

**Table 1:** The three modes used to carry out the tests of our MR system on healthy users: description and working conditions.

Mode	Description	Tracking	Opening degree		
			LH in VR	Exoskeleton	Real RH
M1	Users follow a predefined opening degree with RH, LH is mirrored	RH	Mirrored	Predefined (open-loop)	Following
M2	A target profile defines the LH opening degree, users follow with their RH	RH	Predefined	Predefined (open-loop)	Following
M3	Users move RH arbitrarily, LH is mirrored	RH & LH	Mirrored	Mirrored (closed-loop)	Leading

**Figure 3:**
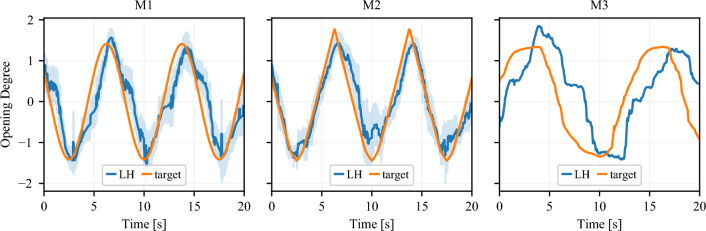
Measured opening degree of LH (blue), and predefined profile (orange) vs. time. The target is given by: a sine function in M1 (left), the animation of the virtual LH in M2 (center); by the arbitrary motion of the users’ RH in M3 (left). Plots for M1 and M2 report the average (line) and standard deviation (shaded area) over all users since the target is the same for all runs; the plot of M3 reports the run for a single user. For the sake of clarity, the plots refer to a 20 s extract of the experiments.

The evaluation through the System Usability Scale (SUS) over all the modes and users yields an average score of 72.14, with a standard deviation of 11.54, which is above 70 and thus rates the usability of our system as acceptable^32^. Out of the 21 scores, only 4 are between 70 and 60, corresponding to a marginal level of usability^32^. Only one score is lower (32.5) and might be symptomatic of concern^32^; however, it shall be noted that, in the corresponding experiment, the tool malfunctioned and a complete reboot of the headset was necessary, inducing the user to give a low score. The evaluation of the Simulator Sickness Questionnaire (SSQ)^33^ yields an average of 15.31 (out of a maximum value of 235.62) and a standard deviation of 11.88. Such a value might be considered indicative of some degree of sickness^33^. However, the original interpretation of the SSQ has been largely criticized^34–36^. Recent literature confirms that most users who report SSQ scores similar to ours consider the experience more than acceptable^37^. Indeed our participants did not raise any concerns regarding discomfort or sickness during the experiments, as expected since our setup does not involve any discrepancy between visual and vestibular senses^38^.

The system’s components manage to follow the motion commands: both the visualization of the hand motion and the actuation of the exoskeleton work in a satisfactory way. The desired motion is in general followed with good accuracy, as can be qualitatively evaluated in the plots of Fig. 3 (left and center) showing the average opening degree of the users’ LH while following the target profile (visualized as a sine function in VR for M1 and hand animation for M2). This aspect is quantitatively confirmed by the Normalized Cross-Correlation (NCC) between the LH opening degree and the target profile, which is 0.76 for M1 and 0.84 for M2. A NCC value equal to 1.0 indicates a perfect match, and values above 0.5 are indicative of a strong correlation. Furthermore, the position of the maximum of the cross-correlation between the target profile and the measured opening degree signals quantifies the temporal lag between the former and the latter; this indicates the delay that the system achieves when actuating the exoskeleton to guide LH to track the desired opening degree. This delay corresponds to 0.53 s for M1 and 0.21 s for M2. Note that this analysis cannot be carried out for M3 as there is no predefined target to follow, but LH has to follow RH which, in turn, is moved by users at their own pace. We show an example of LH and RH motion during one M3 execution in Fig 3, right. The average NCC between LH and RH motions in M3 independently for each user is 0.74, with an average delay of 1.59 s. These high NCC values (i.e. greater than 0.5) quantitatively confirm that all 3 modalities are suitable to enrich the MT setup with sensorimotor feedback. In fact, the exoskeleton is actuated in such a way that the LH position is close to the target with a negligible delay in M1 and M2. In particular, M2 emerges as the most promising setup. The low value of delays is demonstrative that what the users see in VR well matches the real motion of the hand. This match is an important prerequisite for achieving a satisfactory sense of embodiment of our tool. Indeed, these considerations are in line with the results obtained with the embodiment questionnaire.

**Figure 4:**
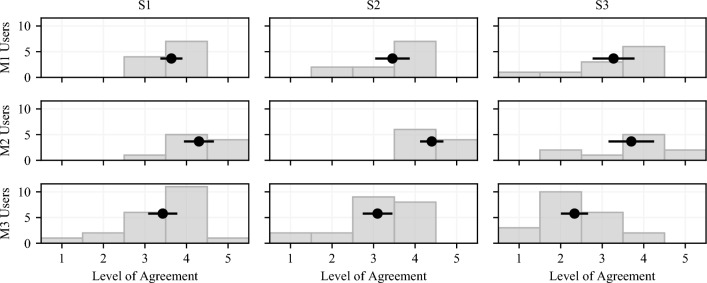
Average (black dot) and standard deviation (black line) of the scores given by the participant to the sense of embodiment questionnaire. The gray histograms in the background indicate the number of participants who gave each answer the same score.

The system’s sense of embodiment is evaluated with a custom questionnaire asking if the virtual LH was felt as the real one (S1); if the motion of the actual LH was perceived as the virtual one (S2); and if the exoskeleton was moving fast enough (S3). Figure 4 summarizes the answers given by respondents using a Likert scale, which ranges from 1 (strongly disagree) to 5 (strongly agree); 3 means neutral. We quantify the sense of embodiment as the average of the scores given to each statement of the questionnaire. M2 yields the highest level of embodiment. We argue that this result is due to the fact that the exoskeleton is directly controlled by the same target profile used to move the virtual LH. Therefore, the delay in the actuation is minimized with a beneficial effect on the difference between what the users see in VR and what they perceive in the real world. In M1, instead, even if the exoskeleton is actuated by a predefined motion as in M2, users focus their attention on the visualization of the sine profile, which is an element of distraction that reduces the sense of embodiment. We observe that M2 scores better than M3 for all the S1 ($p=0.009$), S2 ($p=0.0003$) and S3 statements ($p=0.001$); M2 scores better than M1 for S1 ($p=0.022$) and S2 ($p=0.008$), whereas the comparison is inconclusive for S3 ($p=0.17$).

## Discussion

The proposed MT system builds on mature, commercialized, and inexpensive VR tools, and a simple hand exoskeleton. The simplicity of the exoskeleton implies both drawbacks and advantages. The disadvantages stem from the limited actuation abilities (in terms of speed and force) of our exoskeleton. Indeed, we could not expect high velocity, or high force, performed by our exoskeleton. Therefore we design the M1 and M2 modes in compliance with these limitations in order to guarantee good tracking performance, low actuation delay, and a good sense of embodiment. On the other hand, the simplicity of our exoskeleton implies other factors that are beneficial to the MT context. Firstly, the device is easy to wear and comfortable to use. Secondly, it is soft and compliant, meeting the needs of stroke patients affected by hand rigidity and spasticity. Thirdly, it uses affordable and portable components, favoring widespread use. These aspects make our system a promising candidate to become a domestic therapy tool. This has also important implications for the effectiveness of the MT, as a higher dosage means better therapy outcomes. Furthermore, our tool produces a high sense of embodiment, which provides users with an immersive experience unaffected by external distractions.

The system is well accepted by the users, who perceive it as usable and do not report any sickness due to its utilization. Indeed, since our setup only requires little motion of the hands, and the user to stay seated, there is no perceivable discrepancy between the visual and vestibular senses^38^. This aspect is important to mitigate the simulator sickness and let the users accept the tool so that they are more and more motivated to use it. At the same time, a large use allows designers to collect more feedback to improve the technology and favor its widespread adoption.

The system is lightweight and portable, both in hardware and in the kind of therapy that can be autonomously performed by patients with little or no infrastructure and assistance. More expensive and complex systems might guarantee better results; however, our solution proposes a valuable trade-off between devices’ costs and performance. As such, the proposed system can be considered as a complementary or alternative method to unburden the healthcare systems; it could be considered by insurance companies to provide customers with cheaper alternatives. Furthermore, an affordable and portable rehabilitation tool could represent a solution for lower-middle-income countries, which are highly hit by the cost of stroke disabilities.

Future development will be devoted to including the multi-user utility of the VR headset, opening interesting paths in the telemedicine domain. In this way, in fact, other actors could remotely participate in the same VR therapy scenario. For example, a therapist could connect to the rehabilitation session of a patient and offer them remote assistance and supervision. The tests that we carried out with healthy users are promising. Overall, M2 results in the best mode to maximize acceptability, usability, and sense of embodiment of the proposed MT setup. The promising results pave the road towards the deployment of such a system in a clinical trial involving stroke patients, to validate the therapeutic efficacy of the proposed tool in real clinical scenarios. These activities will require a long-term plan, involving a large number of patients, classified according to the specificity of their impairment, balanced according to their gender and experience with the used technology, and a historical analysis of the therapeutic efficacy. In parallel, a health technology assessment will be carried out to verify the regulatory aspects as well as the social and economic impact of our tool.

## Conclusion

We have designed and developed a MT system based on VR and actuated exoskeleton technology. We have designed different working modes and tested them with healthy subjects, aiming at finding the best therapeutic setup. Our analysis confirms that our system is usable, accepted by users, and provides a good sense of embodiment. The tool has been shown to be promising for future tests with patients. Furthermore, it opens interesting possibilities in the field of telemedicine and modern rehabilitation procedures, with ethical, social, and economic repercussions.

## Methods

### Procedure

The 21 participants are divided into two groups: the first (composed of 11 users) performed M1 first and then M3; the remaining 10 participants execute M2 first and then M3. Each of them is asked to sit at a desk. They wear the exoskeleton at the left hand, then the headset. When the VR application starts, the position of the desk is calibrated according to the user size. Users stay with their elbows on the desk and their hands in front of the headset (see Fig. 2).

The procedure was approved by the local ethics committee of the University of Applied Sciences and Arts of Southern Switzerland (SUPSI) and was performed in accordance with relevant ethical guidelines. Subjects provided informed consent prior to the beginning of the experiment. The subject identifiable in the images and in the video of the experiments gave his consent to publish identifying information or images.

Before each experiment, the participants are given a short explanation about the therapy mode and a few seconds to try the system and get accommodated. Finally, the experimental session actually starts as well as the recording of the hand joint values and VR scene, for plotting and analysis purposes. Each user experiment consists of the execution of two modes (M1 and M3 for 11 participants, M2 and M3 for the remaining 10). Each mode lasts 2 minutes. In M1 and M2 the users are asked to follow the target profile, whereas in M3 they are tasked to perform simple opening and closing movements with RH. The embodiment questionnaire is filled by the users at the end of each mode, whereas SUS and SSQ are filled at the end of each experiment.

### Experimental setup

We have developed a VR application in Unity^39^, a video game engine providing realistic virtual environments, for the Meta Quest 2 headset^40^. This device has four onboard infrared cameras, which point at the workspace of the users’ hands. Such cameras are used by the internal software development kit of the headset to provide hand-tracking capabilities, with no need for any other external sensory infrastructure. This Meta Quest 2 functionality is particularly advantageous for our purposes since it prevents inconvenient cabling, lending our rehabilitation device lightness and portability.

Regarding the exoskeleton, we use the device provided by Emovo Care^41^, a portable and lightweight hand orthosis composed of two exoskeletal fingers that are worn coupling the index with the middle finger, and the ring finger with the pinkie, respectively. Both tendons are actuated by the same motor assisting simple opening and closing motions. Emovo Care’s device enables lightweight, comfortable, and compliant interactions with users’ hands, and has been validated with both healthy users and motor-impaired users. For more technical specifications, the interested reader can refer to the description of its early prototype^42^.

The VR scene is composed of a plain office environment (see Fig. 2) with a PC monitor placed on top of a desk. Users sit in front of the monitor, with their torso orthogonal to a white line running across the desk, which serves as a reference for the users. Thanks to the tracking capabilities of the VR headset, RH is visualized in the VR scene as it moves in reality. Thus, the wrist pose of the virtual LH is mirrored from the pose of RH w.r.t. the white reference line. According to the different modes, the opening degree of LH is controlled according to a predefined motion (in M1 and M2) or mirrored from RH (in M3). At the same time, the exoskeleton is actuated to impose the opening degree of the virtual LH on the users’ real LH.

The signed difference between the opening degree of the mirrored RH and that of the real LH is used as error feedback for a simple proportional law that computes the opening/closing commands sent to the exoskeleton, whose objective is to zero the difference between the virtual and real LH. Such a simple controller is used to implement the closed-loop behavior of the exoskeleton used in M3. The opening degree of the hands is computed as the average values of the pitch angles (that are representative of the opening motion) for each finger joint, excluding the thumb (as it is not actuated by the exoskeleton).

During the experiment, several mechanisms guarantee the user’s safety. At the software level, our implementation provides 3 different safety stops: (*i*) by pressing a keyboard button from the Unity interface; (*ii*) by taking the headset off, which can be detected by a proximity sensor embedded into the VR device; (*iii*) by hiding the RH in order to stop the hand tracking. All these actions will cause the immediate stop of the exoskeleton movement. At the hardware level, the exoskeleton is equipped with two safety mechanisms: (*i*) the exoskeletal fingers have end-stops that prevent the fingers from over-extending or over-flexing; (*ii*) the exoskeletal motion transmission acts as a mechanical fuse, breaking upon failure of all the other safety measures, and preventing any force from being applied to the user any longer.

### Evaluation

We have used two standard questionnaires to evaluate the usability of the system (SUS) and its potential sickness effect (SSQ). Instead, for evaluating the sense of embodiment, we have built a custom questionnaire drawing inspiration from a standardized questionnaire^43^. The statistical analysis of the different modes with respect to the sense of embodiment is performed with a Wilcoxon rank-sum test. The null hypothesis is that the sets of scores given by users to two modes (M2-M1; M2-M3) are drawn from the same distribution. The alternative hypothesis is that values in the first sample are more likely to be larger than the values in the second sample.

The synchronization between the opening degree of the hand wearing the exoskeleton and the target is evaluated by computing the NCC. Also, considering the position of the maximum value for the NCC, we quantify the temporal lag between the two signals.

### Supplementary Information

Supplementary Video 1.

## Data Availability

All data generated or analysed during this study are available upon request to the corresponding author.
